# Nickel Nanoparticles Promote Lung Adenocarcinoma Progression via CDK1-Mediated Fatty Acid Metabolism Regulation

**DOI:** 10.3390/ijms262110624

**Published:** 2025-10-31

**Authors:** Rui-Ze Wu, Bo Zhang, Han-Nong Yu, Qian-Qian Sun, Wen-Xue Yao, Wei-Yang Liu, Jun-Jie Lv, Zhi-Wei Xu, Hong-Qing Qi, Yao Fu, A-Yang Zhao, Yu-Lin Pan, Yong-Hui Wu, Rui Xin

**Affiliations:** 1 Department of Occupational Health, Public Health College, Harbin Medical University, 157 Baojian Road, Harbin 150086, China; 2023020176@hrbmu.edu.cn (R.-Z.W.); 202401069@hrbmu.edu.cn (B.Z.); yuhannong7@163.com (H.-N.Y.); s1208594458@163.com (Q.-Q.S.); yaowenxue158666@163.com (W.-X.Y.); 2024020157@hrbmu.edu.cn (W.-Y.L.); 15942752635@163.com (J.-J.L.); xzw414160446@163.com (Z.-W.X.); 15982678026@163.com (H.-Q.Q.); 102311@hrbmu.edu.cn (Y.F.); panyulin@hrbmu.edu.cn (Y.-L.P.); 2 School of Medicine and Health, Harbin Institute of Technology, 92 Xidazhijie, Harbin 150001, China; 202001434@hrbmu.edu.cn

**Keywords:** nickel nanoparticles, lung adenocarcinoma, fatty acid metabolism, CDK1, carcinogenesis

## Abstract

Nickel nanoparticles (NiNPs) are extensively used in nanotechnology, electronics, and biomedical fields, raising concerns about their pulmonary toxicity and potential role in inducing lung adenocarcinoma (LUAD). While heavy metals, like arsenic and cadmium, are well-known to drive LUAD through metabolic reprogramming, the molecular mechanism linking NiNPs to LUAD—particularly their impact on fatty acid metabolism (FAM)—remains unclear. This study is the first to explore whether NiNPs promote LUAD progression via the CDK1/STAT3/FASN axis, a key regulator of FAM, and to evaluate the natural compound apigenin (API) as a potential inhibitory agent. When human (A549) and mouse (LLC) LUAD cells were exposed to NiNPs, assessments of cell function and protein expression revealed increased malignant phenotypes, including enhanced proliferation, migration, invasion, and epithelial–mesenchymal transition (EMT), along with activation of the CDK1/STAT3/FASN axis and upregulation of FAM-related markers. Genetic silencing of either CDK1 or FASN reversed the dysregulation of FAM and reduced the malignant characteristics of the cells. Molecular docking analysis confirmed that API binds strongly to CDK1, and further experiments demonstrated that API suppresses NiNP-induced tumor growth both in laboratory cell models and in living organisms, while also blocking the activity of the CDK1/STAT3/FASN axis.

## 1. Introduction

Nickel nanoparticles (NiNPs) are nanoscale nickel particles known for their high catalytic activity and stability [1]. With the rapid development of nanotechnology—driven by core principles, such as the quantum size, surface, small-size, and macroscopic quantum tunneling effects, NiNPs have found broad applications in areas including renewable energy, electronics, and magnetic resonance imaging [2,3,4,5]. Nevertheless, as an emerging nanomaterial, their occupational exposure risks and safety guidelines are still under investigation [6]. ISO/TS 27687:2016 lacks specific NiNP limits because NiNPs are emerging nanomaterials, and their toxicological data remain insufficient for formulating standards [7]. Classified as a “respiratory sensitizer” by Germany’s MAK Commission—meaning they can provoke an immune response in the airways and cause allergic reactions upon repeated exposure—NiNPs warrant engineering controls (e.g., local ventilation) and personal protective equipment (e.g., N95 respirators) to minimize exposure [8]. Long-term occupational exposure to NiNPs, as seen in nickel refinery workers, is associated with a significantly increased risk of lung and nasal cancers [9,10]. NiNPs can bypass the upper respiratory tract barrier and deposit in the alveolar cavity. Phagocytosed by alveolar epithelial cells and macrophages, they induce reactive oxygen species (ROS), triggering cell membrane damage, mitochondrial dysfunction, and gene mutations to cause early lung injury [11]. ROS further activate the NF-κB pathway, stimulating the release of pro-inflammatory cytokines (TNF-α, IL-6) that recruit inflammatory cells and establish a self-sustaining inflammatory loop. This persistent inflammation promotes epithelial–mesenchymal transition (EMT) in alveolar cells [12]. Concurrently, activated fibroblasts differentiate into myofibroblasts, secreting and depositing collagen in the lung interstitium to form fibrotic foci, ultimately leading to irreversible pulmonary fibrosis [13]. The cumulative impact of these effects markedly elevates the risk of lung cancer. Despite this, NiNPs have not yet been classified as carcinogens by the International Agency for Research on Cancer (IARC). Thus, systematic studies are imperative to unravel their tumor-promoting potential and molecular mechanisms.

Emerging evidence indicates that NiNP-induced lung adenocarcinoma (LUAD) displays unique features compared to tumors caused by conventional nickel compounds. Due to their small size and high specific surface area, NiNPs accumulate preferentially in alveolar regions and penetrate epithelial barriers more effectively than larger particles [14]. Unlike typical carcinogens that mainly induce genetic mutations, NiNP-driven malignant transformation is accompanied by profound metabolic alterations that are resistant to antioxidant or DNA repair interventions [15]. Our preliminary data show that NiNP exposure induces abnormalities in fatty acid metabolism (FAM) in A549 lung adenocarcinoma cells. FAM is a crucial process for maintaining cellular homeostasis [16]. In lung cancer, cancer cells exhibit marked metabolic reprogramming, being particularly reliant on FAM to support their malignant proliferation [17]. They upregulate fatty acid uptake and synthesis by activating relevant genes and signaling pathways to support rapid growth [18,19]. This metabolic shift not only supplies energy and biosynthetic building blocks but also drives malignancy by modulating oncogenic signaling, positioning FAM as a promising therapeutic target in lung cancer [20]. However, current research has largely focused on intrinsic tumor metabolic dysregulation. The contribution of exogenous factors, such as NiNPs to FAM reprogramming during LUAD onset, remains unexplored. Specifically, the mechanisms by which NiNPs disrupt FAM and the potential involvement of specific signaling axes represent a significant knowledge gap.

Cyclin-dependent kinase 1 (CDK1) is a master regulator of the cell cycle [21]. It acts as a critical checkpoint controller, precisely coordinating cell growth, DNA replication, and mitosis [22]. Recent studies implicate CDK1 in the modulation of FAM [23]. In particular, CDK1 helps maintain triglyceride homeostasis by regulating metabolic enzymes involved in triglyceride synthesis and breakdown [24]. CDK1 overactivation also enhances phosphorylation of signal transducer and activator of transcription 3 (STAT3) at Ser727, increasing its expression [25]. Phosphorylation-activated STAT3 can recognize the promoter region of the Fatty Acid Synthase (FASN) gene via its DNA-binding domain to regulate its methylation status, and recruit the co-activator p300/CBP complex to bind to stable STAT3, thereby sustaining the activation of FASN transcription [26,27,28,29]. As a key rate-limiting enzyme in de novo fatty acid synthesis, FASN regulates intracellular fatty acid biosynthesis and storage. Aberrant FASN expression disrupts fatty acid metabolic homeostasis, triggers lipid dysmetabolism, and contributes to pathological processes including tumor proliferation and energy reprogramming by supplying abnormal lipid substrates, thereby impacting metabolic microenvironmental homeostasis [30]. To date, no studies have reported whether CDK1 participates in NiNP-induced FAM abnormalities.

Apigenin (API), a naturally occurring flavonoid with multiple biological activities [31,32], has recently emerged as a potent antitumor agent. It suppresses tumor cell proliferation and metastasis by modulating ROS, inducing cell cycle arrest, and promoting apoptosis and autophagy [33]. Moreover, API specifically inhibits CDK1/2 activity, resulting in G2/M phase arrest in tumor cells [34]. Based on this evidence, we hypothesize that API may mitigate NiNP-induced pulmonary FAM dysregulation mediated by CDK1.

To address these questions, we will use CCK-8, wound healing, and Transwell assays to assess proliferation, migration, and invasion; qPCR and Western blot to examine CDK1/STAT3/FASN pathway activity and FAM markers, including Acyl-CoA Oxidase 1 (ACOX1), Acetyl-CoA Carboxylase 1 (ACC1), Cluster of Differentiation 36 (CD36). SiRNA-mediated knockdown of CDK1/FASN will confirm pathway dependency. Molecular docking will be employed to identify API as a high-affinity CDK1 ligand. In vivo, a mouse xenograft model will be established, combining acute/subacute NiNP exposure via airway instillation with oral API treatment to monitor tumor growth, histopathology, and molecular profiles. This work aims to elucidate the oncogenic role of NiNPs via the CDK1/STAT3/FASN-FAM axis, propose API as a targeted intervention, and provide a scientific foundation for nanomaterial safety and lung adenocarcinoma prevention.

## 2. Results

### 2.1. Effects of NiNPs on LUAD Cell Growth

**Figure 1:**
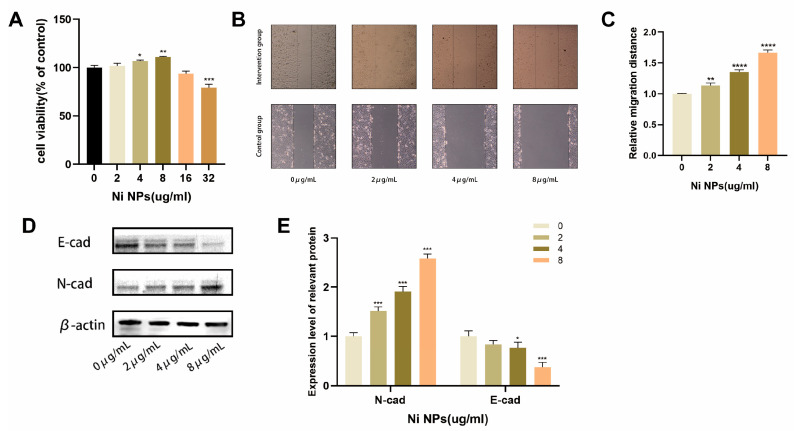
Proliferation, migration, and invasion of A549 cells exposed to NiNPs. (**A**) Cell viability at varying NiNPs doses; (**B**,**C**) cell migration; (**D**,**E**) expression of EMT markers (E-cadherin, N-cadherin). (* *p* < 0.05, ** *p* < 0.01, *** *p* < 0.005, **** *p* < 0.001 vs. 0 group).

To clarify the impact of NiNP exposure on the proliferation ability of LUAD cells, this study used the A549 cell line as the research subject and conducted a CCK-8 assay (Figure 1A). The results revealed that when the concentration of NiNPs reached 2 μg/mL or higher, LUAD cells exhibited a linear growth trend, with peak proliferation observed at 8 μg/mL. Beyond 8 μg/mL, the growth-promoting effect gradually declined. Based on these findings, subsequent experiments utilized exposure concentrations of 2 μg/mL, 4 μg/mL, and 8 μg/mL. The scratch wound healing assay and WB analysis further confirmed (Figure 1B–E) that the migratory capacity and EMT of LUAD cells exhibited a dose-dependent increase with escalating concentrations of NiNPs. Collectively, these results indicate that NiNP exposure enhances malignant biological behaviors in LUAD cells in vitro.

### 2.2. NiNPs Induce Abnormal FAM in LUAD Cells

**Figure 2:**
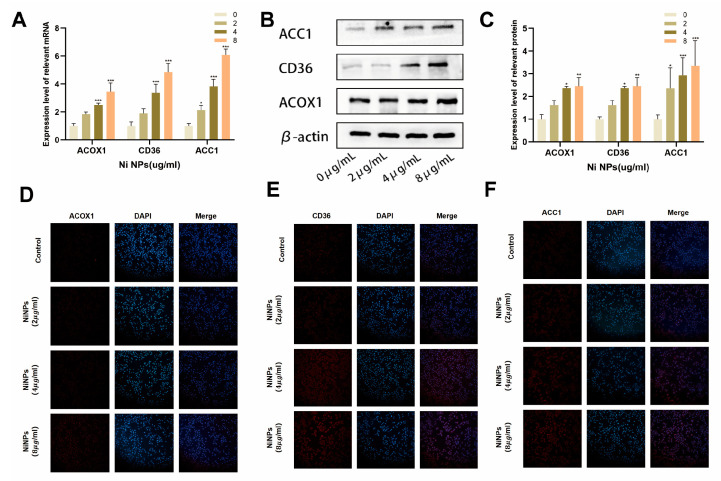
Detection of FAM-related genes/proteins in NiNP-exposed A549 cells. (**A**) FAM-related gene expression; (**B**,**C**) protein levels of ACOX1, CD36, ACC1; (**D**–**F**) immunofluorescence of ACOX1, CD36, ACC1 proteins. (* *p* < 0.05, ** *p* < 0.01, *** *p* < 0.005 vs. 0 group).

To determine whether NiNPs affect FAM in LUAD cells, we analyzed the expression of FAM-related genes in A549 cells after NiNP exposure (Figure 2A). The results showed that NiNPs upregulated the mRNA levels of ACOX1, ACC1, and CD36 in a significant dose-dependent manner. Western blot and immunofluorescence (IF) assays were subsequently employed to validate FAM-related protein expression (Figure 2B–F). Protein expression patterns aligned with mRNA trends, confirming that NiNP exposure disrupts FAM in A549 cells.

### 2.3. NiNPs Activate the CDK1 Pathway

**Figure 3:**
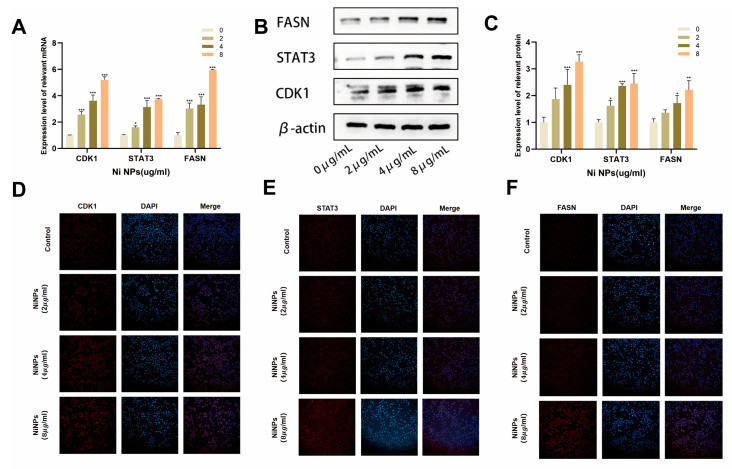
Detection of CDK1 pathway genes/proteins in NiNP-exposed A549 cells. (**A**) CDK1 pathway gene expression; (**B**,**C**) protein levels of CDK1, STAT3, FASN; (**D**–**F**) immunofluorescence of CDK1, STAT3, FASN proteins. (* *p* < 0.05, ** *p* < 0.01, *** *p* < 0.005 vs. 0 group).

Our previous studies identified CDK1 as a critical risk factor in the development of LUAD and found it closely associated with FAM. To determine whether NiNPs influence CDK1 expression, we analyzed mRNA levels of CDK1 and its downstream pathway molecules STAT3 and FASN in A549 cells after NiNP exposure (Figure 3A). RT-qPCR results demonstrated dose-dependent upregulation of CDK1, STAT3, and FASN. Western blot and immunofluorescence (IF) assays further confirmed elevated protein expression of these molecules (Figure 3B–F), suggesting that NiNP-induced FAM dysregulation may result from CDK1 activation.

### 2.4. CDK1 Inhibition Attenuates NiNP-Induced FAM Dysregulation

**Figure 4:**
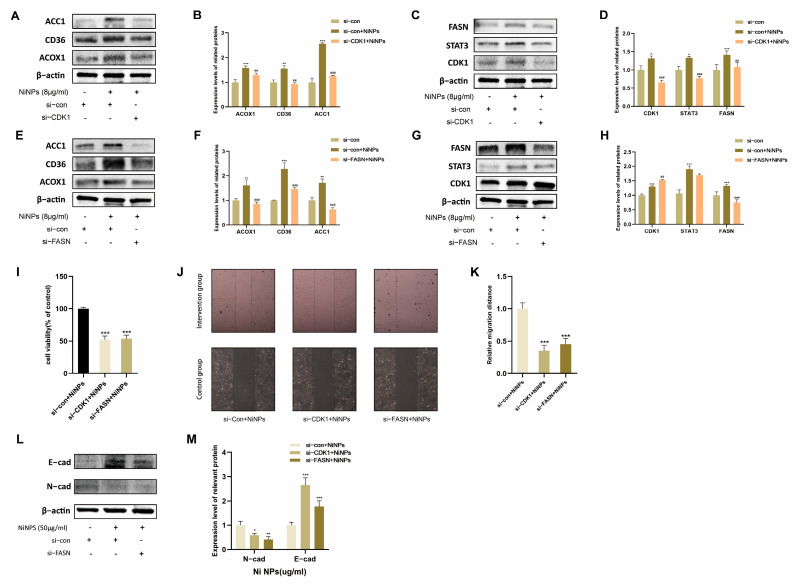
Effects of CDK1 on NiNP-induced FAM in A549 cells. (**A**,**B**) FAM-related proteins after CDK1 silencing; (**C**,**D**) CDK1 pathway proteins after CDK1 silencing; (**E**,**F**) FAM-related proteins after FASN silencing; (**G**,**H**) CDK1 pathway proteins after FASN silencing; (**I**) cell viability after CDK1/FASN silencing + NiNP exposure; (**J**,**K**) cell migration; (**L**,**M**) EMT markers (E-cadherin, N-cadherin). (* *p* < 0.05, ** *p* < 0.01, *** *p* < 0.005 vs. si−con group. ^##^ *p* < 0.01, ^###^ *p* < 0.005 vs. si-con + NiNPs group).

To investigate whether NiNPs affect FAM via CDK1, we silenced CDK1 in A549 cells using siRNA and exposed them to 8 μg/mL NiNPs. Protein levels of STAT3 and FASN were significantly reduced in CDK1-silenced cells (Figure 4A–D). Concurrently, the expression and functional states of key FAM-related proteins were restored to near-normal levels. These findings suggest that CDK1 mediates NiNP-induced FAM dysregulation by regulating downstream pathway molecules.

### 2.5. CDK1 Promotes LUAD Progression via FAM

To assess whether NiNP-induced CDK1 activation drives LUAD progression through FAM, we suppressed FASN in A549 cells and exposed them to 8 μg/mL NiNPs. Suppression of FASN did not alter the expression of its upstream genes, CDK1 and STAT3, but restored FAM-related protein expression (Figure 4E–H). CCK-8 assay, scratch wound healing assay, and WB further revealed that normalization of FAM levels significantly attenuated NiNP-induced proliferation, migration, and EMT in A549 cells (Figure 4I–M).

### 2.6. API Reverses NiNP-Induced FAM Dysregulation

**Figure 5:**
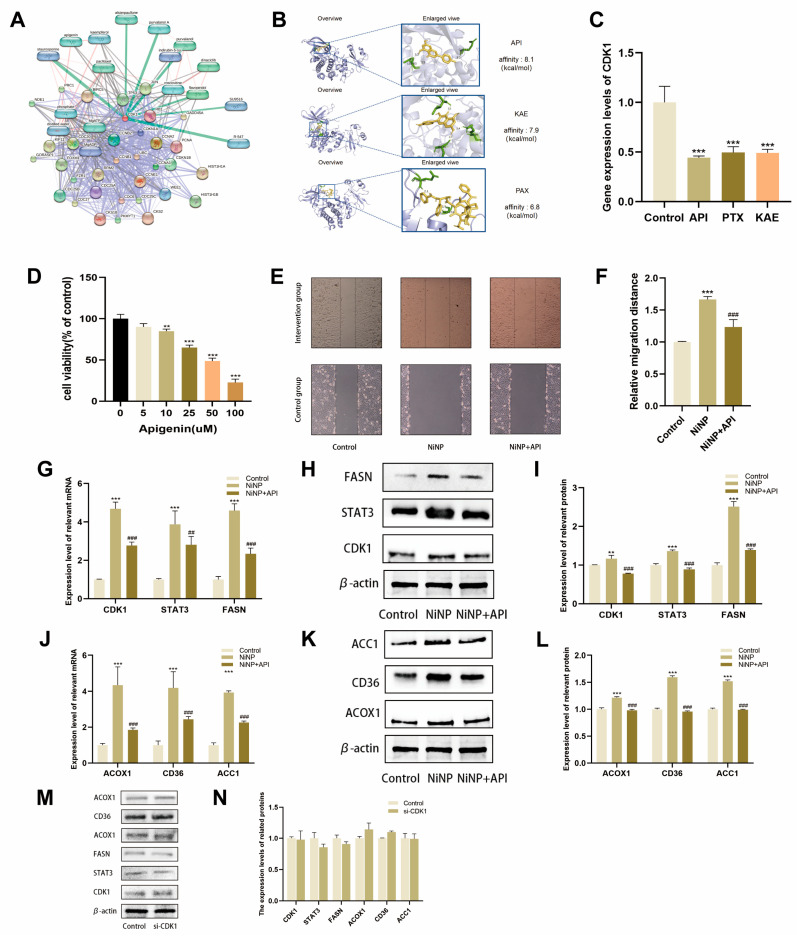
Effects of API on NiNP-induced FAM dysregulation. (**A**) Predicted CDK1 compound-protein interaction network; (**B**) affinity between CDK1 and plant-derived compounds; (**C**) CDK1 mRNA levels in A549 cells pretreated with plant-derived compounds; (**D**) cell viability after apigenin pretreatment + NiNP exposure; (**E**,**F**) migration levels of A549 cells; (**G**) expression of CDK1 pathway-related genes; (**H**,**I**) protein-level expression of CDK1 pathway components; (**J**) expression of FAM-associated genes; (**K**,**L**) protein-level expression of FAM-related markers; (**M**,**N**) impact of CDK1 silencing on API’s protective effects. (** *p* < 0.01, *** *p* < 0.005 vs. Control group. ^##^ *p* < 0.01, ^###^ *p* < 0.005 vs. NiNP group).

Using the STITCH database, we constructed a chemical–protein interaction network and identified the top three natural plant compounds with relevance to CDK1: apigenin (API), paclitaxel (PTX), and kaempferol (KAE). Molecular docking simulations were performed to screen the most effective CDK1 inhibitor. A549 cells were pretreated with these compounds 2 h before NiNP exposure, and CDK1 expression was assessed. API exhibited the strongest inhibitory effect (Figure 5A–C). Subsequent CCK-8 and scratch assays confirmed that API reversed NiNP-induced enhancements in A549 cell proliferation and migration (Figure 5D–F). qPCR and Western blot analyses further demonstrated dose-dependent reversal of CDK1 pathway and FAM-related gene/protein expression (Figure 5G–L). To verify API’s dependency on CDK1, CDK1-silenced cells were pretreated with API. The protective effects of API were significantly diminished in CDK1-deficient cells (Figure 5M–N), indicating that API primarily acts through CDK1.

### 2.7. NiNPs Promote LUAD Progression via CDK1/FAM In Vivo

**Figure 6:**
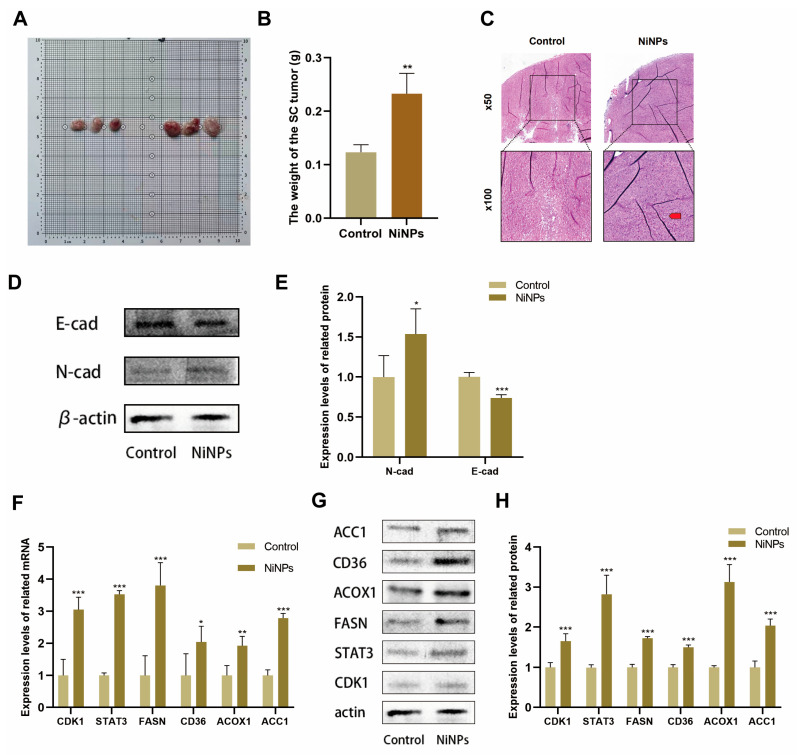
Acute NiNP exposure induces tumor pathological changes and FAM abnormalities in mice. (**A**) Tumor morphology post-intervention. Left: control group; Right: NiNPs group (3 mice/group, representative tumors); (**B**) tumor weight; (**C**) tumor organizational structure changes (red arrows indicate the NiNPs group has significant tissue lesions); (**D**,**E**) EMT-related protein expression in tumors; expression of FAM-related and CDK1 pathway genes (**F**) and proteins (**G**,**H**) in tumors. (* *p* < 0.05, ** *p* < 0.01, *** *p* < 0.005 vs. Control group).

To further validate the results of the in vitro cell experiments, in this study, male C57BL/6 mice were used to establish a subcutaneous tumor xenograft model for in vivo research. During the seven-day acute and 28-day subacute exposure periods, general clinical signs of mice in each group were monitored daily, and no mouse deaths were observed. Mice in the NiNPs group exhibited significant abnormalities, while none were observed in the control group, specifically reduced spontaneous activity, dull hair, and greater weight loss in the NiNPs group. The results showed that, compared with the control group, the weights of the transplanted tumors in the mice of the group with acute NiNP exposure significantly increased (Figure 6A,B). In terms of histological analysis, the results of hematoxylin-eosin (HE) staining were also presented (Figure 6C). These results showed that the acute NiNP exposure group exhibited significant pathological alterations. Cells showed marked pleomorphism, hyperchromatic and irregular nuclei with increased mitotic figures, and disrupted architectural organization. The tumor parenchyma lost its regular glandular structures, with cells arranged in a disorganized, invasive pattern. In contrast, control group tissues displayed normal cellular morphology and well-preserved tissue architecture, with intact glandular arrangements and minimal atypia. Western blot analysis further showed a significant upregulation of EMT in tumors (Figure 6D,E).

As detected by Western blot and qPCR techniques (Figure 6F–H), NiNPs could significantly alter the expression of FAM-related proteins in tumor tissues in vivo. Meanwhile, the CDK1/STAT3/FASN signaling pathway was activated, and the changing trends of the expressions of related molecules were highly consistent with the results of the in vitro cell experiments. These findings further provided a complete set of experimental evidence both in vitro and in vivo for revealing the tumor-promoting mechanism of NiNPs.

### 2.8. API Reverses NiNP-Induced LUAD Progression In Vivo

**Figure 7:**
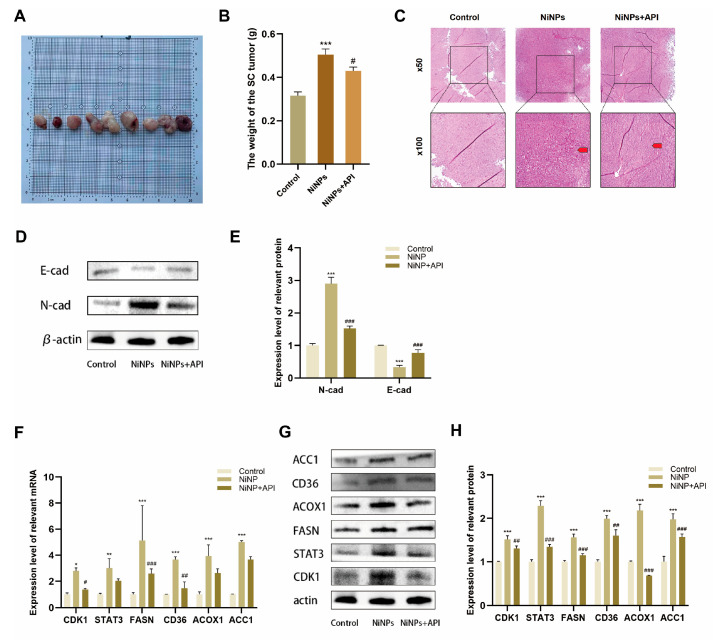
API reverses NiNP-induced tumor pathological changes and FAM abnormalities in mice. (**A**) Tumor morphology post-intervention. Left to right: control, NiNPs, NiNPs + API groups (3 mice/group, representative tumors); (**B**) tumor weight; (**C**) tumor organizational structure changes (red arrows indicate the NiNPs group has significant tissue lesions, while the NiNPs+API group shows marked improvement); (**D**,**E**) EMT-related protein expression in tumors; (**F**–**H**) expression of FAM-related and CDK1 pathway genes (**F**) and proteins (**G**,**H**) in tumors. (* *p* < 0.05, ** *p* < 0.01, *** *p* < 0.005 vs. Control group. ^#^ *p* < 0.05, ^##^ *p* < 0.01, ^###^ *p* < 0.005 vs. NiNP group).

In order to explore the role of API in the in vivo environment, we also carried out interventions on C57BL/6 mice. During the 28-day intervention period, general clinical signs of mice in each group were also monitored daily, and no mouse deaths were observed. The clinical signs of mice in the NiNPs + API group were significantly improved compared with those in the NiNPs group, specifically characterized by normalized responsiveness to stimuli, restored hair luster, and daily food intake that recovered to a level close to that of the control group. Subsequently, we found that API intervention significantly reduced tumor weight compared to the NiNP-exposed group (Figure 7A,B). Histopathological analysis demonstrated (Figure 7C) that tumor cells exhibited reduced pleomorphism, with nuclei showing less hyperchromatism and more regular contours, and mitotic figures (including atypical ones) were significantly decreased. Measurements of EMT marker proteins further demonstrated that apigenin reverses the EMT process (Figure 7D,E).

Western blot and qPCR confirmed that API normalized FAM-related protein expression and suppressed CDK1/STAT3/FASN pathway activation (Figure 7F–H). These in vivo results, spanning histomorphology, protein, and gene expression, confirm that API mitigates NiNP-driven LUAD progression, offering critical insights for therapeutic development.

## 3. Discussion

This study provides the first evidence that NiNPs promote LUAD initiation and progression by activating CDK1, which upregulates STAT3, enables recognition of the FASN gene promoter, sustains FASN transcription, and ultimately leads to FAM dysregulation. We further show that the natural compound API effectively counteracts this pathway through specific targeting and inhibition of CDK1. Experimentally, NiNP exposure significantly enhanced LUAD cell proliferation, migration, and EMT, accompanied by upregulated expression of key FAM-related molecules. API reversed these abnormalities in a dose-dependent manner, an effect strictly dependent on CDK1 inhibition. These results not only establish a metabolic mechanism for NiNP-induced cancer progression but also identify the CDK1-mediated axis as a novel regulatory pathway and API as its corresponding protective agent, suggesting a potential targeted strategy for managing nanomaterial-related lung cancer.

Using CCK-8, wound healing, and Transwell invasion assays, we observed concentration-dependent increases in proliferation, migration, and invasion following NiNP exposure, helping set appropriate doses for subsequent mechanistic studies. Concurrent measurements revealed elevated mRNA and protein levels of core FAM regulators—ACOX1, ACC1, and CD36—with increasing NiNP concentrations. This effect is closely tied to NiNP-induced ROS production, which drives metabolic dysfunction and DNA damage [35,36], key drivers in carcinogenesis. ACOX1 upregulation may stabilize β-catenin via palmitoylation, activating proliferation-related signaling [37]; high ACC1 expression inhibits fatty acid oxidation by producing malonyl-CoA, helping maintain tumor energy homeostasis [38]; and CD36 activation enhances uptake of exogenous long-chain fatty acids and promotes EMT and angiogenesis through PPARγ and NF-κB pathways [39]. Together, these molecules create a permissive metabolic environment for NiNP-induced LUAD progression, suggesting FAM dysregulation as a central mechanism. While FAM abnormalities are often viewed as intrinsic to cancer cells and driven by genetic or signaling defects [40,41,42,43], our work demonstrates that exogenous nanoparticles can actively trigger this metabolic reprogramming, directly linking an environmental exposure to tumor metabolic alterations and opening new avenues for studying pollutant-induced carcinogenesis.

Our prior work established CDK1 as linked to both FAM abnormalities and poor prognosis in LUAD patients [44], motivating the investigation of its role in NiNP-induced metabolic reprogramming. Here, we found that NiNP exposure robustly activates the CDK1/STAT3/FASN axis, paralleling FAM disruption and increased malignant phenotypes. As the rate-limiting enzyme in de novo lipogenesis, FASN overexpression supplies abundant lipids to support rapid tumor growth [28], identifying FAM as a critical downstream effector of NiNPs. This finding substantially expands the functional repertoire of CDK1. Traditionally recognized as a core cell cycle regulator controlling division and proliferation [21], we now show that under NiNP exposure, CDK1 also promotes FASN transcription via STAT3 [29,45], contributing to metabolic reprogramming in a context-specific manner. Importantly, our focus on CDK1’s metabolic role does not contradict its classical cell cycle function—the two pathways may act synergistically to promote NiNP-driven LUAD.

To identify intervention strategies, we screened the STITCH database and performed AutoDock Vina docking, identifying API as a high-affinity CDK1 binder, superior to other candidates such as PTX and KAE. In comparative intervention experiments, API outperformed both: PTX, an anti-microtubule agent, showed only 49% CDK1 inhibition and required high, toxic doses; KAE, although less toxic and structurally similar, had weaker CDK1 binding and inhibition. We, therefore, selected API for further study. This natural flavonoid has documented anticancer activity in lung, breast, prostate, and colon cancers [46,47,48,49]. Here, API dose-dependently reversed NiNP-induced proliferation, migration, and FAM dysregulation in LUAD cells. Crucially, CDK1 silencing abrogated API’s protective effects, confirming that its action depends specifically on CDK1 targeting. This adds a new dimension to API’s known antitumor mechanisms—which include reducing oxidative stress via the Nrf2/NF-κB pathway [50,51] or modulating lipid accumulation via XO/NLRP3 [52]—by showing that it also normalizes FAM reprogramming via direct CDK1 binding, supporting its development as a chemopreventive agent for nanomaterial-associated lung cancer.

In summary, through systematic in vitro and in vivo experiments, this study reveals a novel mechanism by which NiNPs drive LUAD, i.e., disrupting FAM homeostasis by activating the CDK1/STAT3/FASN signaling axis, while confirming that API can reverse this process by targeting CDK1. The innovative value of this research lies in establishing a direct link between exogenous nanomaterial exposure and tumor metabolic reprogramming, breaking the traditional cognition that FAM abnormalities are only intrinsic characteristics of tumor cells; clarifying CDK1 as a core regulatory factor connecting nanomaterial exposure and metabolic disorder, expanding its biological functions; discovering a new mechanism by which API regulates FAM through targeting CDK1, providing a new direction for the application of natural compounds; and finally, through consistent evidence from in vitro and in vivo experiments, offering important theoretical basis and experimental support for the safety control of nanomaterials and the prevention and treatment of LUAD.

This study has limitations. We tested only 20–100 nm NiNPs, leaving smaller, more reactive particles unexamined; we did not validate direct molecular interactions (e.g., CDK1-STAT3 binding by CoIP) in the proposed axis or dissect synergy among FAM-related factors (ACOX1/ACC1/CD36); and we used high-dose airway instillation and subcutaneous tumors in mice, which do not fully recapitulate human chronic low-dose inhalation or the native lung microenvironment. Future studies should include a range of NiNP sizes, verify direct binding events, investigate FAM network interactions, and implement chronic inhalation or orthotopic models.

## 4. Materials and Methods

### 4.1. Cell Lines and Culture

A549 cells (Chinese Academy of Sciences, Beijing, China) and LLC mouse LUAD cells (Zhejiang Baidi Biotechnology Co., Hangzhou, China) were cultured in DMEM containing 10% fetal bovine serum and 1% penicillin-streptomycin. Frozen cells were rapidly thawed in a 37 °C water bath, seeded into culture flasks, and incubated at 37 °C with 5% CO_2_. When confluence reached 80–90%, cells were washed with PBS, digested with 0.25% trypsin, and passaged at a 1:3–1:4 ratio using fresh medium, with identical protocols for both cell lines [53,54].

### 4.2. Preparation of NiNPs Suspension

NiNPs (20–100 nm) were purchased from Aladdin Biochemical Technology Co., Ltd. (Shanghai, China). Briefly, 1 mg of NiNPs was accurately weighed using an analytical balance and transferred to a sterile centrifuge tube. Subsequently, 1 mL of phosphate-buffered saline (PBS) was added, and the mixture was sonicated for 30 min to ensure homogeneous dispersion. The prepared suspension was stored at 4 °C and used within 24 h to maintain stability [11].

### 4.3. CCK-8 Assay

Cell viability was assessed using the CCK-8 assay. Cells were seeded in 96-well plates at a density of 5 × 10^3^ cells/well. After complete adhesion, 10 μL of CCK-8 reagent was added to each well, followed by incubation at 37 °C with 5% CO_2_ for 1.5 h. Absorbance at 450 nm was measured using a microplate reader, and cell viability percentages were calculated relative to the control group.

### 4.4. Wound Healing Assay

Cell migration was analyzed using a wound healing assay. Cells were cultured in 6-well plates until full confluence. A sterile pipette tip was used to create a scratch in the monolayer. After washing with PBS to remove detached cells, serum-free medium was added. Wound closure was observed and recorded at 0 and 24 h post-scratch using an inverted microscope.

### 4.5. siRNA Transfection for Gene Silencing

Cells were seeded in 6-well plates at 2 × 10^5^ cells/well and cultured to 60–70% confluence. Target siRNA and control siRNA were diluted in serum-free medium and mixed with transfection reagent to form complexes after 15 min of incubation at room temperature. The complexes were then added to the cells and incubated at 37 °C with 5% CO_2_ for 24 h.

### 4.6. Real-Time qPCR

Total RNA was extracted using an RNA extraction kit (R711–01, Nanjing Novizan Biotechnology Co., Ltd., Nanjing, China) according to the manufacturer’s instructions. RNA concentration and purity were determined using a spectrophotometer. cDNA was synthesized from 1 µg of total RNA using a reverse transcription kit (R412–01, Nanjing Novizan Biotechnology Co., Nanjing, China). Amplification was performed with SYBR Green Master Mix on a real-time PCR system. Details of primer design are shown in Table A1.

### 4.7. Western Blot Analysis

Samples were lysed on ice for 40 min using lysis buffer containing 1% PMSF. The lysates were centrifuged at 12,000× *g* for 15 min at 4 °C to collect supernatant proteins. Proteins were denatured by boiling at 100 °C for 10 min, separated by SDS-PAGE, and transferred to PVDF membranes. Membranes were blocked with rapid blocking buffer for 10 min, incubated with primary antibodies (A11420, A11216, A19050, A19627, A5792, A21217, A20798, A0433, ABclonal, Wuhan, China) overnight at 4 °C, followed by secondary antibodies (AS014, ABclonal, Wuhan, China) at 37 °C for 1.5 h. Finally, ECL substrate was used for signal detection, and images were captured using a chemiluminescence imaging system.

### 4.8. Chemical–Protein Interaction Analysis

Potential interactions between CDK1 and plant-derived compounds were explored using the STITCH database. CDK1 was set as the core target protein with species limited to Homo sapiens and an interaction confidence threshold of 0.7. Plant-origin small molecules were screened to construct a CDK1-compound interaction network. Visualization tools were employed to identify potential regulatory relationships.

### 4.9. Molecular Docking Prediction

AutoDock Vina 1.1.2 was used to simulate interactions between target compounds and receptor proteins. Receptor proteins were prepared using AutoDock Tools 1.5.6 by removing water molecules, adding polar hydrogens, and assigning Gasteiger charges. Ligand structures were optimized with PyMOL 2.5. Docking parameters included a grid box covering the entire binding pocket and default exhaustiveness value of 8.

### 4.10. Animal Experiment Design

Six-week-old male C57BL/6 mice (Liaoning Changsheng Biotechnology Co., Liaoning, China) were acclimatized for one week. LLC mouse LUAD cells (5 × 10^5^ cells, Baidi Biotechnology Co., Zhejiang, China) were subcutaneously implanted to monitor tumor formation for one week [55]. Mice were then randomly divided into groups (n = 9/group):

Acute control (7 days): Administered 50 μL saline via airway instillation twice weekly.

Acute NiNP exposure (7 days): 50 μL 200 μg/mL NiNPs (10 μg/mouse per dose, total 20 μg/7 days) via airway instillation twice weekly.

Subacute control (28 days): Same as acute control for 4 weeks.

Subacute NiNP exposure (28 days): Same as acute NiNP exposure for 4 weeks (80 μg NiNPs/mouse total) [11].

API protective group (28 days): 1.25 g/kg API via oral gavage daily (starting 1 day pre-NiNPs), with NiNPs treatment as subacute exposure [56].

Mice were euthanized by cervical dislocation under isoflurane anesthesia. Transplanted tumors were dissected, weighed, and stored (part fixed in 4% paraformaldehyde for pathology; rest at −80 °C for Western blot/qPCR).

This experiment was approved by the Experimental Animal Ethics Committee of Harbin Medical University (Approval No.: HMUIRB-2023-045), complying with Guidelines for Ethical Review of Experimental Animals (GB/T 35892-2018).

### 4.11. Hematoxylin and Eosin (HE) Staining

Tissue samples were fixed in 4% paraformaldehyde (PFA) at 4 °C overnight, then dehydrated through a graded ethanol series (70%, 80%, 95%, 100%) for 1 h each. Samples were cleared in xylene (two changes, 1 h each) and embedded in paraffin wax (melting point 58–60 °C) using a tissue embedding center. The embedding process included two cycles of paraffin infiltration (2 h each), followed by rapid cooling at 4 °C to solidify the blocks [57]. Sections (4–6 μm) were deparaffinized, rehydrated, and stained with hematoxylin for 5–10 min. Differentiation was performed with 1% acid alcohol, followed by bluing in tap water for 10 min. Eosin staining (1–3 min) was conducted before dehydration, clearing, and mounting. Tissue morphology was observed under a light microscope.

### 4.12. Statistical Analysis

The data were statistically analyzed using GraphPad Prism 9.0 or SPSS software 26.0. Normality and homogeneity of variance tests were first conducted. When the conditions were met, an independent samples *t*-test was used for comparisons between two groups, and for multiple groups, one-way analysis of variance (ANOVA) followed by Tukey’s test was applied. All experimental results were repeated three times, and a *p* value < 0.05 was considered to indicate a statistically significant difference [58].

## 5. Conclusions

This study elucidates the mechanism by which NiNPs promote LUAD progression via CDK1-mediated FAM reprogramming and the protective role of API. In vitro and in vivo models show that NiNPs activate the CDK1/STAT3/FASN axis to disrupt FAM homeostasis, conferring metabolic advantages for tumor cell proliferation and migration. Database-based screening combined with molecular docking simulations identifies API as a specific CDK1 inhibitor reversing NiNP-induced FAM dysregulation and tumor progression, clarifying NiNPs’ oncogenic potential and proposing a CDK1/FAM-targeted LUAD therapy with natural compounds.

## Data Availability

The original contributions presented in the study are included in the article and Supplementary Materials; further inquiries can be directed to the corresponding authors.

## Supplementary Materials

The following supporting information can be downloaded at: https://www.mdpi.com/article/10.3390/ijms262110624/s1.

## Abbreviations

The following abbreviations are used in this manuscript:

NiNPs	Nickel Nanoparticles
LUAD	Lung Adenocarcinoma
FAM	Fatty Acid Metabolism
CDK1	Cyclin-Dependent Kinase 1
STAT3	Signal Transducer and Activator of Transcription 3
FASN	Fatty Acid Synthase
API	Apigenin
IARC	International Agency for Research on Cancer
ROS	Reactive Oxygen Species
PPARγ	Peroxisome Proliferator-Activated Receptor γ
NF-κB	Nuclear Factor Kappa-B
Nrf2	Nuclear Factor Erythroid 2-Related Factor 2
XO	Xanthine Oxidase
NLRP3	NOD-Like Receptor Pyrin Domain-Containing 3
ACOX1	Acyl-CoA Oxidase 1
ACC1	Acetyl-CoA Carboxylase 1
CD36	Cluster of Differentiation 36
EMT	Epithelial–Mesenchymal Transition
PC-TWA	Permissible Concentration–Time Weighted Average
ISO	International Organization for Standardization
MAK	Maximale Arbeitsplatzkonzentration
GBZ	Guobiao Zhi (Chinese National Occupational Health Standard)

## Appendix A

**Table A1:** Primer sequence and number of base pairs for verified gene expression.

CDK1	AAACTACAGGTCAAGTGGTAGCC	23
	TCCTGCATAAGCACATCCTGA	21
STAT3	ACCAGCAGTATAGCCGCTTC	20
	GCCACAATCCGGGCAATCT	19
FASN	AAGGACCTGTCTAGGTTTGATGC	23
	TGGCTTCATAGGTGACTTCCA	21
ACOX1	AATCGGGACCCATAAGCCTTT	21
	GGGAATACGATGGTTGTCCATTT	23
ACC1	ATGTCTGGCTTGCACCTAGTA	21
	CCCCAAAGCGAGTAACAAATTCT	23
CD36	TACTCTTGCCTTGCGGACTAA	21
	CCGAAGCAGTTCACCGATATAC	22
