# Supplementary material for: Nickel Nanoparticles Promote Lung Adenocarcinoma Progression via CDK1-Mediated Fatty Acid Metabolism Regulation
Source: Int J Mol Sci. 2025 Oct 31;26(21):10624. doi: 10.3390/ijms262110624 (PMC12611017; doi:10.3390/ijms262110624)
Supplement: Supplementary file 1 [file ijms-26-10624-s001.zip › ijms-3947151-supplementary.pdf]

1. Nickel nanoparticles (NiNPs), an emerging environmental nanomaterial, promote lung adenocarcinoma (LUAD) progression by disrupting fatty acid metabolism (FAM), with a clear dose-dependent effect on enhancing malignant phenotypes (proliferation, migration, EMT) of LUAD cells.

2. This study first identifies that NiNPs activate the CDK1/STAT3/FASN signaling axis to induce FAM dysregulation (upregulating ACOX1, ACC1, CD36), establishing a direct link between environmental nanomaterial exposure and tumor metabolic reprogramming.

3. The natural compound apigenin (API) specifically targets CDK1 to reverse NiNPs-induced FAM disruption and LUAD progression, validated both in vitro and in vivo, offering a potential strategy for mitigating nanomaterial-associated carcinogenesis.

4. Findings provide critical insights into the environmental health risks of NiNPs, supporting the need for improved nanomaterial safety standards, and highlight CDK1 as a therapeutic target for environment-related lung cancer.
